# Draft genome sequences of a novel *Escherichia coli* K-12 MG1655 L-form strain and its evolved variant

**DOI:** 10.1128/mra.01283-24

**Published:** 2025-05-27

**Authors:** Marjolein E. Crooijmans, Johannes H. de Winde, Dennis Claessen

**Affiliations:** 1 Institute of Biology, Leiden University, Sylviusweg, Leiden, Netherlands; University of Maryland School of Medicine, Baltimore, Maryland, USA

**Keywords:** L-form, *Escherichia coli*

## Abstract

L-forms are bacterial variants capable of proliferating without a cell wall. In this evolution study, we present draft genome sequences of a novel *Escherichia coli* K-12 L-form strain and an evolved variant that exhibits enhanced growth. These draft sequences provide insights into the genetic adaptations associated with cell wall-independent growth.

## ANNOUNCEMENT

L-forms are bacteria that proliferate without a cell wall often due to unique, species-specific mutations (1–3). These cells are crucial for studying bacterial survival under cell wall-targeting antibiotics and provide insights into bacterial persistence and antibiotic resistance. To investigate mutations that enable L-form growth, we generated an *Escherichia coli* K-12 MG1655 L-form strain from a Weizmann Institute parental strain (WT) carrying a kanamycin-resistant green fluorescent protein (GFP) reporter plasmid under control of the serW promoter (4). The L-form strain (L-form^0^) was created by diluting the WT overnight culture in L-phase broth with 50 µg mL^−1^ kanamycin and 0.4 mg mL^−1^ penicillin (5), then incubating it at 37°C. A 1 mL sample (8 × 10 ^ 8 spheroplasts) was plated on L-phase media agar with horse serum and Iberian agar, then exposed to UV light for 1 min. After 72 h at 30°C, a mucoid colony appeared.

**Fig 1:**
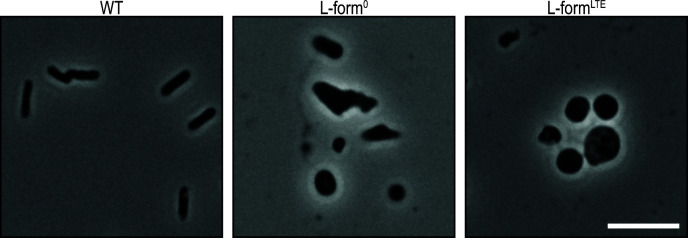
Morphology of the *E. coli* L-form strains. Wild-type (WT) cells were grown overnight in LB medium, while the L-form^0^ and L-form^LTE^ strains were grown in L-phase broth. Brightfield microscope images were captured after placing 5 µL of each culture onto microscope slides. Scale bar: 10 µm.

The L-form^0^ colony was used to inoculate L-phase broth with kanamycin and penicillin (Fig. 1). To enhance growth, we performed a long-term evolution experiment, transferring cells of L-form^0^ weekly into L-phase broth with antibiotics. After 121 weeks, reaching the 800th generation, it was marked L-form^LTE^ (Fig. 1). For genomic analysis, L-form^0^ and L-form^LTE^ were cultured in L-phase broth to exponential phase (OD600 = 0.35) and WT in LB medium (OD600 = 2.0) both at 37°C overnight. DNA was extracted with the GenElute Bacterial Genomic DNA Kit. A minimum of 500 ng DNA at >25 ng µL^−1^ was submitted for Illumina sequencing by BaseClear (Leiden, Netherlands).

The library was prepared with the Genomic Nextera XT Kit, including quality control (QC) and quantification steps. Whole-genome sequencing was performed on Illumina NovaSeq 6000. FASTQ-format reads were quality-filtered to remove PhiX and adaptors, ensuring a minimum read length of 50 bp. Secondary QC was done with FASTQC version 0.11.8 (6).

Reads were imported into Geneious Prime 2025.0.3 (7) and trimmed with BBDuk version 38.84 (trim low-quality min 30, discard short reads 30 bp) (8). WT reads were mapped to the K-12 MG1655 genome (National Center for Biotechnology Information U00096.3, 17 Nov. 2022) to create a WT sequence. Trimmed reads from L-form^0^ and L-form^LTE^ were then mapped to this WT sequence to generate consensus sequences. Annotation used the U00096.3 reference genome (Live Annotate & Predict, 95% similarity). Default parameters were used for all software per Geneious guidelines, unless otherwise specified (July 2024). Notably, we observed that after the formation of L-form^LTE^, a part of the original plasmid was replaced by an *intF*-containing region, which continued to drive the GFP signal. Variation analysis in Geneious (find variations/SNPs with default minimum variation of 0.25, WT as reference) identified SNPs, insertions, and deletions in L-form^0^ and L-form^LTE^ (Table 1) (9). L-form^LTE^ lost over 4,500 bp mainly due to loss of some RAC prophage genes, including *trkG*, *ynaK*, *ydaY*, and *ynaA*, impacting motility and biofilm formation (10). These genomic changes support cell wall-independent growth (9).

**TABLE 1:** General features of the WT, L-form^0^, and L-form^LTE^ genomes

	*E. coli* genomes		
	WT	L-form^0^	L-form^LTE^
BioSample	SAMN47195525	SAMN47195526	SAMN47195527
GenBank chromosome no.	CP184745	CP184743	CP184741
GenBank plasmid no.	CP184746	CP184744	CP184742
SRA no.	SRR30543495	SRR30543494	SRR30543493
Total no. of raw Illumina reads	7,655,618 (3,827,809 paired)	7,157,602 (3,578,801 paired)	4,849,560 (2,424,780 paired)
FASTQC quality control score	35.52	35.59	35.25
Total no. of trimmed Illumina reads	6,303,758 (3,151,879 paired)	5,872,224 (2,936,112 paired)	3,776,720 (1,888,360 paired)
Read length (bp)	50–151	50–151	50–151
No. of contigs	2	2	2
Largest contig size (bp)	4,628,058	4,628,071	4,623,550
Total length (bp)	4,632,732	4,632,792	4,628,271
Coverage of total genome (×)	139	130	81
Genome GC content (%)	50.8	50.8	50.8
No. of chromosomal genes (coding)	4,217	4,214	4,207
No. of mutated chromosomal genes		5	16
Location of mutated genes		*ykgH* (324,865), *infA* (926,290–926,352), *ndh* (1,166,207), *yjbH* (4,225,963), *sltY* (4,617,491)	*lpdA* (128,990), *ykgH* (324,865), *infA* (926,290–926,352), *rpsA* (962,661), *ndh* (1,166,207), *mepM* (1,927,515–1,927,588), *rcsA* (2,010,915), *yfbK* (2,370,803), *ptsI* (2,520,982), *nadK* (2,737,836), *envZ* (3,521,426), *glmU* (3,900,444), *wecB* (3,956,856), *yjbH* (4,225,963), *yjiA* (4,574,924), *sltY* (4,617,491)

## Data Availability

The complete sequences have been deposited in GenBank. The BioProject accession number is PRJNA1230865. The BioSample and GenBank accession numbers are provided in Table 1. The assembled version described in this paper is the first version.
